# Histone Methyltransferases SUV39H1 and G9a and DNA Methyltransferase DNMT1 in Penumbra Neurons and Astrocytes after Photothrombotic Stroke

**DOI:** 10.3390/ijms222212483

**Published:** 2021-11-19

**Authors:** Svetlana Sharifulina, Valentina Dzreyan, Valeria Guzenko, Svetlana Demyanenko

**Affiliations:** Laboratory of Molecular Neurobiology, Academy of Biology and Biotechnology, Southern Federal University, pr. Stachki 194/1, 344090 Rostov-on-Don, Russia; svetlana.shariuflina@gmail.com (S.S.); dzreyan@sfedu.ru (V.D.); vguzenko@sfedu.ru (V.G.)

**Keywords:** ischemic stroke, epigenetics, histone methyltransferase, DNA methyltransferase

## Abstract

Background: Cerebral ischemia, a common cerebrovascular disease, is one of the great threats to human health and new targets for stroke therapy are needed. The transcriptional activity in the cell is regulated by epigenetic processes such as DNA methylation/demethylation, acetylation/deacetylation, histone methylation, etc. Changes in DNA methylation after ischemia can have both neuroprotective and neurotoxic effects depending on the degree of ischemia damage, the time elapsed after injury, and the site of methylation. Methods: In this study, we investigated the changes in the expression and intracellular localization of DNA methyltransferase DNMT1, histone methyltransferases SUV39H1, and G9a in penumbra neurons and astrocytes at 4 and 24 h after stroke in the rat cerebral cortex using photothrombotic stroke (PTS) model. Methods of immunofluorescence microscopy analysis, apoptosis analysis, and immunoblotting were used. Additionally, we have studied the effect of DNMT1 and G9a inhibitors on the volume of PTS-induced infarction and apoptosis of penumbra cells in the cortex of mice after PTS. Results: This study has shown that the level of DNMT1 increased in the nuclear and cytoplasmic fractions of the penumbra tissue at 24 h after PTS. Inhibition of DNMT1 by 5-aza-2′-deoxycytidine protected cells of PTS-induced penumbra from apoptosis. An increase in the level of SUV39H1 in the penumbra was found at 24 h after PTS and G9a was overexpressed at 4 and 24 h after PTS. G9a inhibitors A-366 and BIX01294 protected penumbra cells from apoptosis and reduced the volume of PTS-induced cerebral infarction. Conclusion: Thus, the data obtained show that DNA methyltransferase DNMT1 and histone methyltransferase G9a can be potential protein targets in ischemic penumbra cells, and their inhibitors are potential neuroprotective agents capable of protecting penumbra cells from postischemic damage to the cerebral cortex.

## 1. Introduction

Cerebral ischemia, a common cerebrovascular disease, is one of the great threats to human health [1]. Blockage of cerebral vessels in ischemic stroke (70–80% of all strokes) disrupts blood flow and the supply of oxygen and glucose to surrounding tissues. In the ischemic nucleus nerve cells die fast. Additionally, toxic factors (glutamate, K+, free radicals, acidosis, etc.) spread and damage the surrounding cells and tissues in the following hours [2,3]. During this time (2–6 h, “therapeutic window”), it is possible to save neurons in the transition zone (penumbra), decrease damage, and reduce the neurological consequences of a stroke. However, studies of potential neuroprotectors—calcium channel blockers, excitotoxicity inhibitors, antiapoptotic agents, antioxidants, etc.—which have shown promise in experiments on cell cultures or laboratory animals, have not proven effective protection of the human brain from stroke without unacceptable side effects [4,5,6,7]. The transcriptional activity in the cell is regulated by epigenetic processes such as DNA methylation/demethylation, acetylation/deacetylation, histone methylation/demethylation, histone phosphorylation, etc. DNA methylation is the most well-studied epigenetic modification. DNA methylation involves the attachment of a methyl group to cytosine in the CpG dinucleotide in DNA, where cytosine and guanine are linked by a phosphate group (5′CpG3′). Oxidation of methylated cytosine leads to its demethylation [5,6,7]. DNA methylation does not occur at every cytosine residue in the genome. Only about 60% of all CpG dinucleotides are methylated. If CpG islands (CGIs) in the promoter region of a gene undergo methylation, this usually leads to its suppression. Regulatory regions of many human and animal genes contain unmethylated CpG dinucleotides grouped into CGIs. These are usually promoters of housekeeping genes expressed in all tissues, and in the promoters of tissue-specific genes, CGIs are unmethylated only in those tissues where this gene is expressed [4,5,6,7]. Adding a methyl group to CpG sites can prevent gene transcription in different ways. DNA methylation can directly prevent the binding of DNA-binding factors to transcription sites. In addition, methyl groups in CpG dinucleotides can be recognized by the methyl-CpG-binding domain (MBD) family, such as MBD1–4 and MECP2 [5,6]. The binding of these proteins to methylated CpG sites recruits histone or chromatin-modifying protein complexes, which will assemble a number of complexes that provide repression of gene transcription [4,5,6,7].

At least two methylation systems with different methylases operate in mammalian cells. Methylation de novo is carried out by DNA methyltransferases DNMT3a and DNMT3b, which introduces elements of variability in the methylation profile. Maintenance methylation is provided by DNMT1, which attaches methyl groups to cytosines on one of the DNA strands where the complementary strand is already methylated (accuracy rate > 99%) [8].

Despite the fact that DNA methylation is a fairly common epigenetic modification, the level of DNA methylation in the mammalian genome does not exceed 1%. This is due to the instability of 5-methylcytosine, which undergoes spontaneous deamination to thymine during DNA repair. This results in a rapid depletion of CpG sites in the genome [9]. Nonetheless, DNA methylation is still critical in biological processes such as differentiation, genomic imprinting, genomic stability, and X chromosome inactivation.

DNA methylation and repression of the expression of a number of genes increase in brain cells after ischemia [10,11,12]. The occlusion of the middle cerebral artery (CCA) within 30 min increased the total level of DNA methylation by a factor of four that correlated with the growth of cell damage and neurological deficit. Changes in DNA methylation after ischemia can occur not only at the global level, but also at the promoters of individual genes [13]. These changes can have both neuroprotective and neurotoxic effects depending on the degree of ischemia damage, the time elapsed after injury, and the site of methylation [14,15].

Methylation of histones by histone methyltransferases regulates transcriptional processes in cells. It is known that methylation of lysines 9 and 27 in histone H3, as well as lysine 20 in histone H4, globally suppresses transcription, and methylation of lysine 4 in histone H3 often correlates with transcriptional activity [16]. Lysine methyltransferases SUV39H1 and G9a are best studied histone methyltransferases. SUV39H1 and G9a methylate lysines in histones H3 and H4, leading to the formation of large regions of heterochromatin where gene expression is suppressed. G9a is more responsible for mono and dimethylation of lysine 9 in histone H3 (H3K9Me1 and H3K9Me2), while SUV39H1 is responsible for trimethylation (H3K9Me3) [16,17]. SUV39H1 and G9a are also involved in the repression of individual genes and in a number of neuropathological processes [18]. The damage to peripheral nerves increased the expression of SUV39H1 in the nuclei of neurons in the ganglia of the spinal cord that are involved in the transmission of pain information. This caused nociceptive hypersensitivity. It is assumed that SUV39H1 inhibitors can serve as promising drugs for the treatment of pain hypersensitivity caused by damage to peripheral nerves [18]. Pharmacological inhibition or knockout of histone methyltransferases SUV39H1 or G9a protected cultured neurons from ischemic damage in an oxygen- and glucose-free environment [18,19].

In this study, we investigated the changes in the expression and intracellular localization of DNA methyltransferase DNMT1, histone methyltransferases SUV39H1 and G9a in penumbra neurons and astrocytes in penumbra nuclear and penumbra cytoplasmic fractions at 4 and 24 h after photothrombotic stroke (PTS). In an attempt to find possible neuroprotectors, we studied the effect of DNMT1 and histone methyltransferase G9a inhibitors on the volume of PTS-induced infarction and apoptosis of penumbra cells in the cortex of mice after PTS.

## 2. Results

### 2.1. Expression and Localization of DNA Methyltransferase DNMT1

DNA methylation leading to gene silencing is carried out by DNA methyltransferases DNMT1 (supporting methylation in interphase cells) or DNMT3a and DNMT3b (de novo methylation at early stages of development as well as its changes during cell differentiation). DNMT1 maintains the DNA methylation pattern after cell repair or division. It attaches methyl groups to one of the DNA strands where the complementary strand is methylated. In a normal brain DNMT1 has been detected [20]. However, the role of DNMT in the brain’s response to ischemic injury is poorly understood. It has been shown that the global DNA methylation carried out by methyltransferases DNMT is enhanced 16–24 h after cerebral ischemia and reperfusion, which indicates a decrease in biosynthetic processes [21].

**Figure 1:**
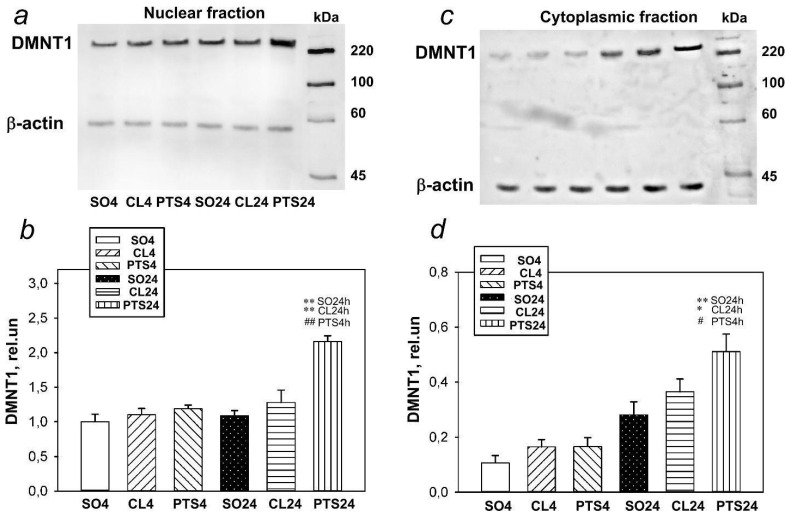
The expression of DNMT1in nuclear (**a**,**b**) and cytoplasmic (**c**,**d**) fractions of penumbra tissue at 4 or 24 h after photothrombotic stroke (PTS) in the rat cerebral cortex. The significant differences * *p* < 0.05 and ** *p* < 0.01 compared to sham-operated animals (SO) or contralateral cerebral cortex of the same animals (CL4 and CL24), # *p* < 0.05 and ## *p* < 0.01 compared to the PTS4 are shown. One-way ANOVA. M ± SEM. *n* = 7. Rel.un—the ratio of the optical density of the strip of the protein studied to the optical density of the strip of the protein load marker (actin).

Western blotting data in our experiments have shown that the level of DNMT1 in both the nuclear and cytoplasmic fractions of the penumbra tissue did not change 4 h after PTS in the rat cerebral cortex, but significantly increased after 24 h (Figure 1).

**Figure 2:**
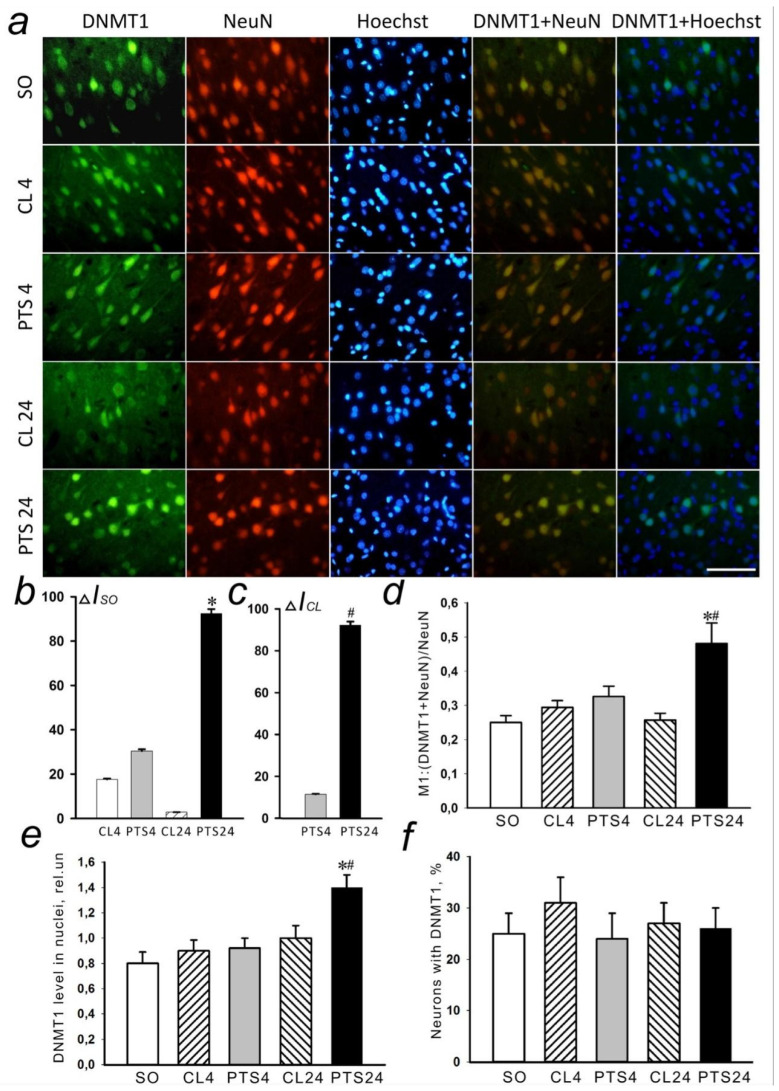
The changes in DNMT1 localization and level in the neurons of ischemic penumbra at 4 and 24 h after photothrombotic stroke (PTS) in the rat cerebral cortex. The significant difference in localization and level of DNMT1 at 4 and 24 h after PTS (PTS4 and PTS24, respectively) is shown compared to the contralateral cortex of the same animals (CL4 and CL24), or the cerebral cortex of sham-operated rats (SO). (**a**) Immunofluorescence of DNMT1 (green), neuronal marker NeuN (red), nuclear chromatin marker Hoechst 33342 (blue), and image overlay. The scale bar is 50 μm. (**b**) Percent changes (ΔISO) of DNMT1 levels in the penumbra (PTS4 or PTS24) and CL4 or CL24 compared to SO at PTS4 or PTS24. (**c**) Percent changes (ΔICL) of DNMT1 levels in the penumbra at PTS4 or PTS24 compared to CL4 and CL24. (**d**) Coefficient M1 of DNMT1 co-localization with the neuronal marker (NeuN) in different control and experimental groups. (**e**) Immunofluorescence of DNMT1 in cell nuclei. (**f**) Percentage of cells expressing DNMT1 per 100 cells stained with Hoechst 33342 * *p* < 0.05 compared to SO, # *p* < 0.05 compared to CL4 and CL24 of the same animals. One Way ANOVA; M ± SEM; *n* = 7.

**Figure 3:**
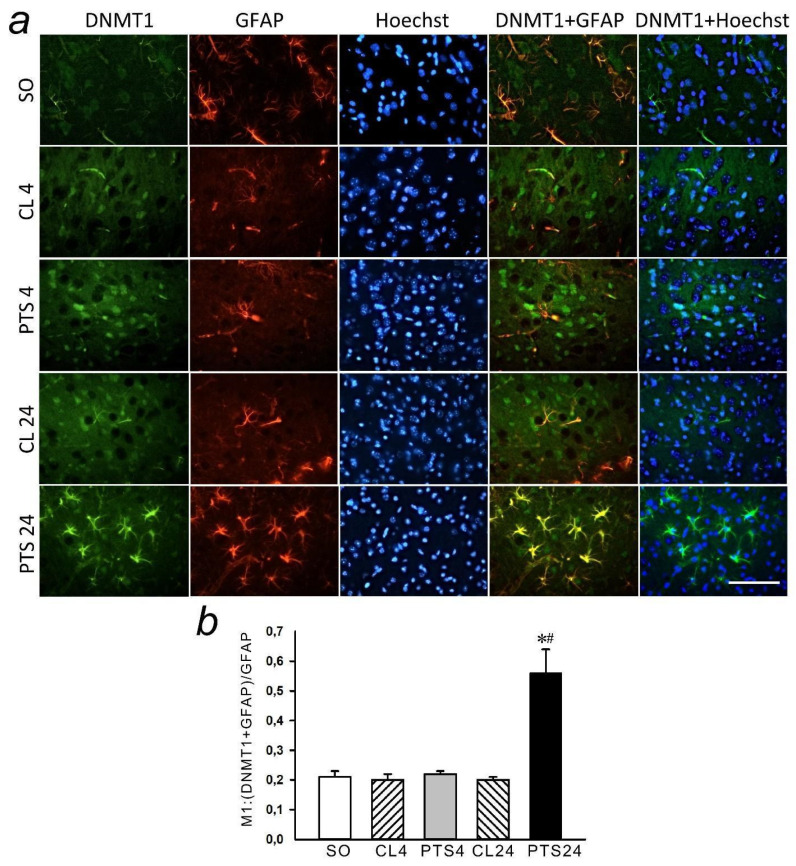
The changes of DNMT1 levels in the astrocytes of ischemic penumbra at 4 and 24 h after photothrombotic stroke (PTS) in the rat cerebral cortex. The levels of DNMT1 at 4 and 24 h after PTS are shown (PTS4 and PTS24, respectively) compared to the contralateral cortex of the same animals (CL4 and CL24), or the cortex of sham-operated rats (SO). (**a**) Immunofluorescence of DNMT1 (green), marker of astrocytes GFAP (red), nuclear chromatin marker Hoechst 33342 (blue), and image overlay. The scale bar is 50 μm. (**b**) Coefficient M1 of DNMT1 co-localization with astrocyte marker (GFAP) in different control and experimental groups. * *p* < 0.05 compared to SO, # *p* < 0.05 compared to CL4 and CL24. One Way ANOVA; M ± SEM; *n* = 7.

These results were confirmed by the data obtained by immunofluorescence microscopy analysis (Figure 2a–c) that have shown that the level of DNMT1 in the penumbra increased (Figure 2b,c) at 24 h after PTS. A colocalization of DNMT1 with markers of neurons NeuN (Figure 2d) and astrocytes GFAP (Figure 3b) significantly increased.

As NeuN selectively labels the nuclei of neurons (Figure 2a), and GFAP stains the bodies of astrocytes, including their processes (Figure 3a), these data explain the results of immunoblotting. The increase in the level of DNMT1 in the nuclear fraction of the penumbra most likely was due to increased expression of neurons in the nuclei, and the increase in the level in the cytoplasmic fraction is due to the expression of this protein in the body and processes of astrocytes.

An increase by 75% (*p* < 0.05) in DNMT1 expression in the nuclei of penumbra cells was shown at 24 h after PTS (Figure 2e). At the same time, there was no increase in the number of cells expressing the protein (Figure 2f).

DNMT1 is a nuclear protein and its appearance in the cytoplasm of astrocytes could be explained by the fact that after synthesis in the cytoplasm the protein must be transported to the nucleus that occurs in neurons. In astrocytes, synthesis of DNMT1 can also be stimulated, but its transport to the nucleus is difficult or much slower than synthesis, so that it accumulates in the cytoplasm. These data are consistent with the results of works published previously [21,22,23]. A similar nuclear-cytoplasmic localization of DNMT1 was observed in reactive astrocytes after traumatic brain injury in contrast to normal localization in the nuclei of neurons [23].

### 2.2. Expression and Localization of SUV39H1 Histone Methyltransferase

**Figure 4:**
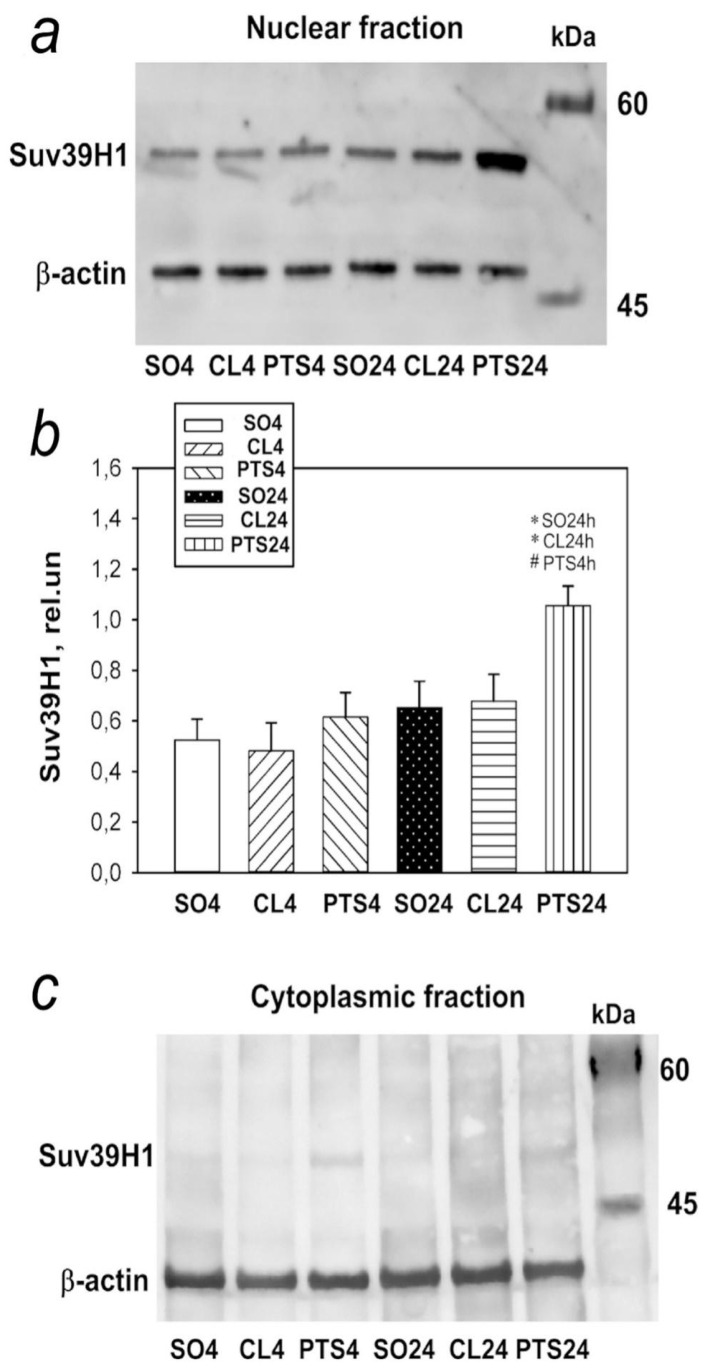
Suv39H1 expression in nuclear (**a**,**b**) and cytoplasmic (**c**) fractions of penumbra tissue at 4 and 24 h after photothrombotic stroke (PTS) in the rat cerebral cortex. The expression of Suv39H1 is shown at 4 and 24 h after PTS (PTS4 and PTS24, respectively) * *p* < 0.05 compared to sham-operated (SO) animals or contralateral cerebral cortex (CL4 and CL24) of the same animals, # *p* < 0.05 compared to PTS4. One-way ANOVA. M ± SEM. *n* = 7. Rel.un—the ratio of the optical density of the strip of the protein studied to the optical density of the strip of the protein load marker (actin).

**Figure 5:**
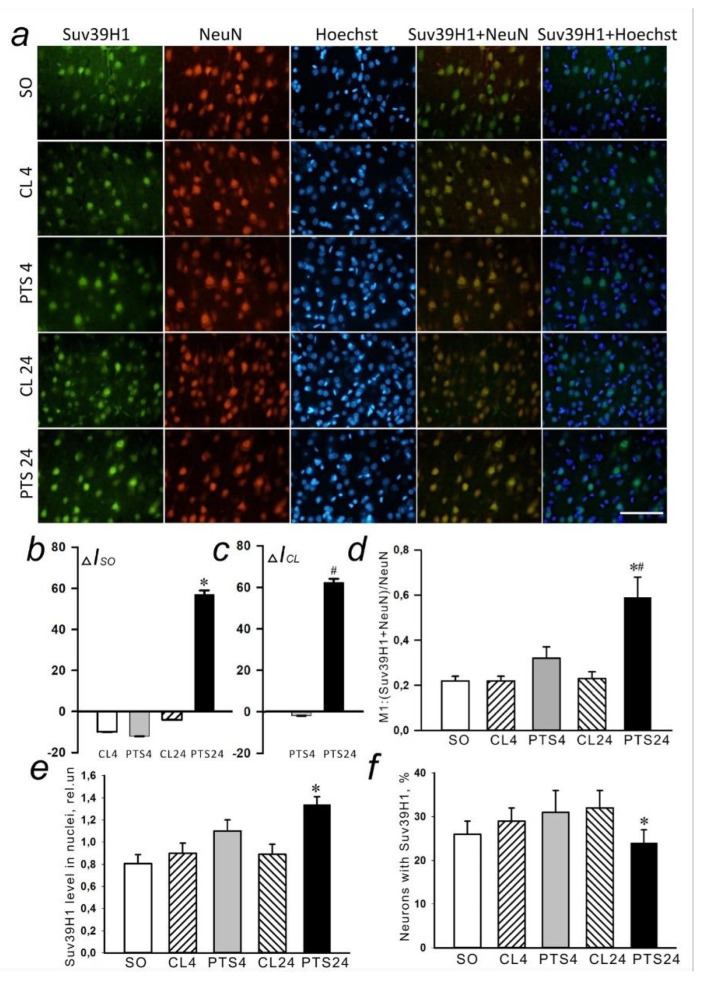
The changes in Suv39H1 localization and levels in the neurons of ischemic penumbra 4 and 24 h after photothrombotic stroke in the rat cerebral cortex. The localisation and level of Suv39H1 are shown at 4 and 24 h after PTS (PTS4 and PTS24, respectively) compared to the contralateral cortex of the same animals (CL4 and CL24), or the cerebral cortex of sham-operated rats (SO). (**a**) Immunofluorescence of Suv39H1 (green), neuronal marker NeuN (red), nuclear chromatin marker Hoechst 33342 (blue), and image overlay. The scale bar is 50 μm. (**b**). Percent changes (ΔISO) of Suv39H1 levels in the penumbra at PTS4 or PTS24 and CL4 or CL24 compared to SO. (**c**) Percent changes (ΔICL) of Suv39H1 levels in the penumbra at PTS4 or PTS24 compared to the CL4 or CL24. (**d**) Coefficient M1 of Suv39H1 co-localization with the neuronal marker (NeuN) in different control and experimental groups. (**e**) Immunofluorescence of Suv39H1 in cell nuclei. (**f**) Percentage of cells expressing Suv39H1 per 100 cells stained with Hoechst 33342 * *p* < 0.05 compared to SO, # *p* < 0.05 compared to theCL4 or CL24. One Way ANOVA; M ± SEM; *n* = 7.

**Figure 6:**
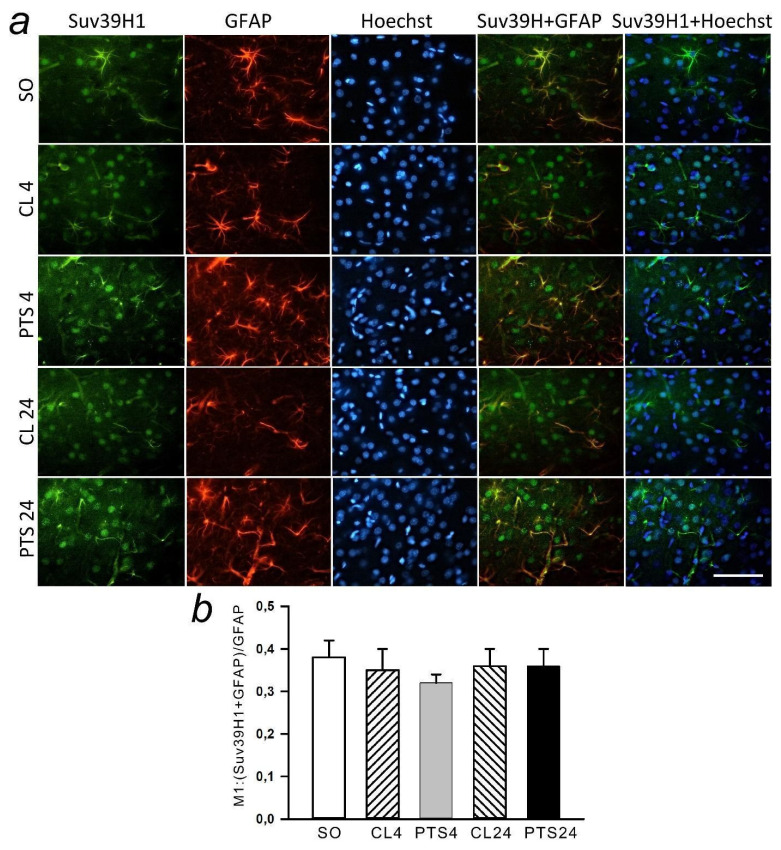
The changes of Suv39H1 levels in the astrocytes of ischemic penumbra at 4 and 24 h after photothrombotic stroke (PTS) in the rat cerebral cortex. The level of Suv39H1 is shown at 4 and 24 h after PTS (PTS4 and PTS24, respectively) compared to the contralateral cortex of the same animals (CL4 and CL24), or the cortex of sham-operated rats (SO). (**a**) Immunofluorescence of Suv39H1 (green), marker of astrocytes GFAP (red), nuclear chromatin marker Hoechst 33342 (blue), and image overlay. The scale bar is 50 μm. (**b**) Coefficient M1 of Suv39H1 co-localization with astrocyte marker (GFAP) in different control and experimental groups. One Way ANOVA; M ± SEM; *n* = 7.

In the present experiments histone methyltransferase SUV39H1 was detected exclusively in the nuclear fraction of the rat cerebral cortex in control samples (Figure 4, Figure 5a and Figure 6a). Its level in the cytoplasm of cells was so low that it was practically not detected by immunoblotting (Figure 4c). The level of SUV39H1 in the nuclear fraction of the penumbra did not change at 4 h after PTS, but it significantly increased at 24 h after PTS (Figure 4a,b).

Immunofluorescence microscopy also revealed an increase in the level of SUV39H1 at 24 h after PTS compared with both control groups (Figure 5a–c). SUV39H1 was localized mainly in the nuclei of neurons (Figure 5a and Figure 6a) and the colocalization of SUV39H1 with neuronal nuclei increased at 24 after PTS (Figure 5d). PTS did not affect colocalization of SUV39H1 with the astrocyte marker GFAP (Figure 6b).

Analysis of SUV39H1 fluorescence in the nucleus of cells revealed an increase by 63% (*p* ≤ 0.05) in the level of protein in the nuclei (Figure 5e) at 24 h after PTS, while the number of cells expressing the protein was even reduced compared to the indicator in the contralateral hemisphere in the same period by 34% (*p* ≤ 0.05) (Figure 5f).

### 2.3. Expression and Localization of Histone Methyltransferase G9a

**Figure 7:**
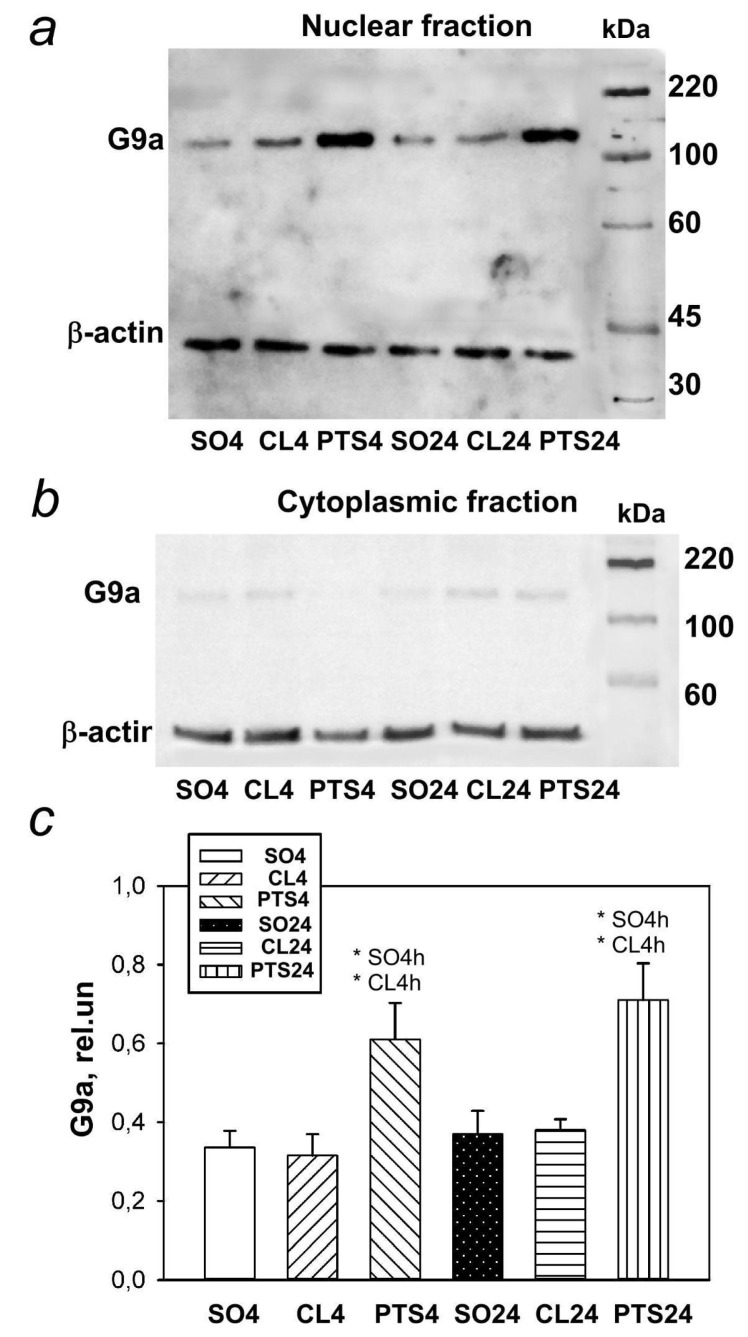
G9a expression in nuclear (**a**,**c**) and cytoplasmic (**b**) fractions of penumbra tissue 4 or 24 h after photothrombotic stroke (PTS) in the cerebral cortex of rats. * *p* < 0.05 compared to sham-operated animals (SO) or contralateral cerebral (CL) cortex of the same animals. One-way ANOVA. M ± SEM. *n* = 7. Rel.un—the ratio of the optical density of the strip of the protein studied to the optical density of the strip of the protein load marker (actin).

The level of histone methyltransferase G9a was also low in the nuclear fraction of control samples of the rat cerebral cortex, and in the cytoplasmic fraction it was practically undetectable (Figure 7b). The G9a level in the penumbra increased at 4 and 24 h after PTS (Figure 7a,c).

**Figure 8:**
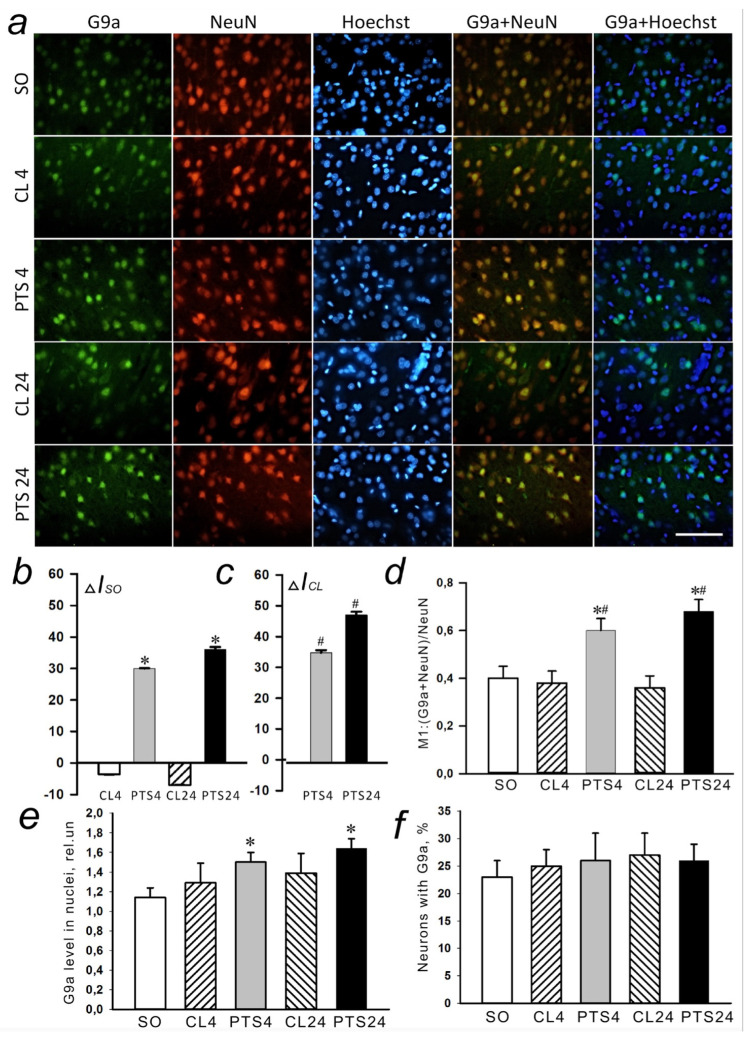
The changes in G9a localization and levels in the neurons of ischemic penumbra at 4 and 24 h after photothrombotic stroke (PTS) in the rat cerebral cortex. The localization of G9a at 4 and 24 h after PTS (PTS4 and PTS24, respectively) compared to the contralateral cortex of the same animals (CL4 and CL24), or the cerebral cortex of sham-operated rats (SO). (**a**) Immunofluorescence of G9a (green), neuronal marker NeuN (red), nuclear chromatin marker Hoechst 33342 (blue), and image overlay. The scale bar is 50 μm. (**b**) Percent changes (ΔISO) of G9a levels in the penumbra (PTS4 or PTS24) and CL4 or CL24 compared to SO. (**c**) Percent changes (ΔICL) of G9a levels in the penumbra (PTS4 or PTS24) compared to CL4 or CL24. (**d**) Coefficient M1 of G9a co-localization with the neuronal marker (NeuN) in different control and experimental groups. (**e**) Immunofluorescence of G9a in cell nuclei. (**f**) Percentage of cells expressing G9a per 100 cells stained with Hoechst 33342 * *p* < 0.05 compared to SO, # *p* < 0.05 compared to CL4 or CL24. One Way ANOVA; M ± SEM; *n* = 7.

**Figure 9:**
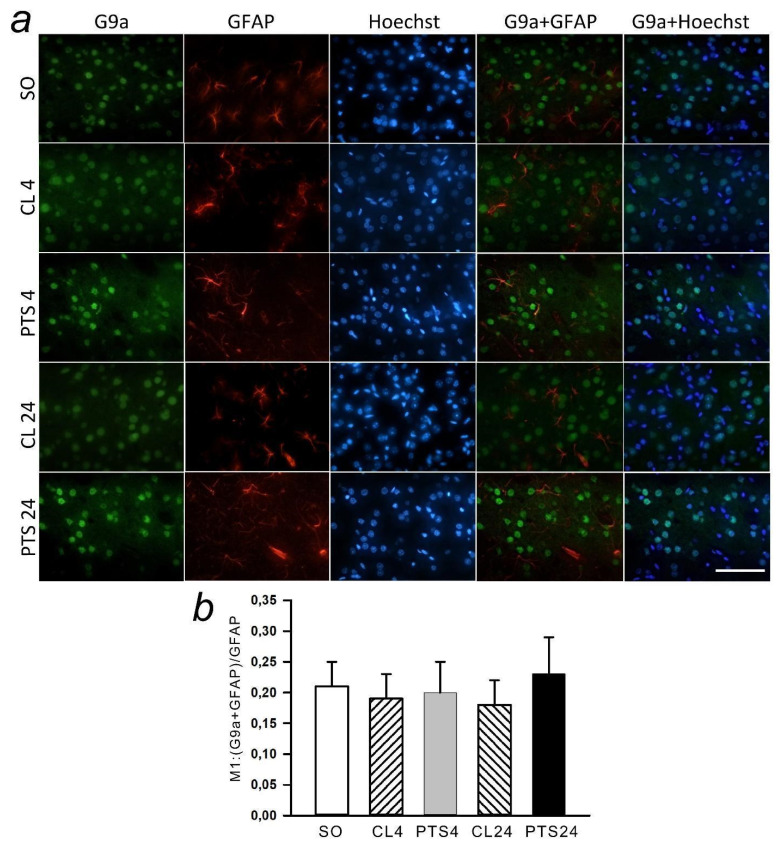
The changes of G9a levels in the astrocytes of ischemic penumbra 4 and 24 h after photothrombotic stroke (PTS) in the rat cerebral cortex. G9a levels (PTS4 and PTS24, respectively) compared to contralateral cortex of the same animals (CL4 and CL24), or the cortex of sham-operated rats (SO) are shown. (**a**) Immunofluorescence of G9a (green), marker of astrocytes GFAP (red), nuclear chromatin marker Hoechst 33342 (blue), and image overlay. The scale bar is 50 μm. (**b**) Coefficient M1 of G9a co-localization with astrocyte marker (GFAP) in different control and experimental groups. One Way ANOVA; M ± SEM; *n* = 7.

Immunofluorescence microscopy revealed exclusively nuclear localization of G9a in neurons (Figure 8a), but not penumbra astrocytes (Figure 9a). The G9a level in the penumbra cells significantly increased relative to the level in the contralateral hemisphere of the same rats (Figure 8b) or in the cerebral cortex of sham-operated rats (Figure 8c) at 4 and 24 h after PTS. This increase can be due to the increased expression of G9a in the nuclei of neurons, as at these time points the coefficient of colocalization G9a with the marker of neuronal nuclei NeuN also increased (Figure 8d), while the colocalization of G9a with the marker of astrocytes GFAP did not change (Figure 9b).

In addition, analysis of G9a fluorescence in the nucleus of cells showed an increase by 32% and 44% in its level in the nucleus at 4 and 24 h after PTS (Figure 8e), respectively, although the number of cells expressing the protein did not increase (Figure 8f).

An increase in the level of SUV39H1 in the penumbra was found at 24 h after PTS, and G9a was overexpressed at 4 and 24 h after PTS.

### 2.4. The Changes of Histone H3 Methylation Level in the Penumbra: H3K9diMe and H3K4Me

**Figure 10:**
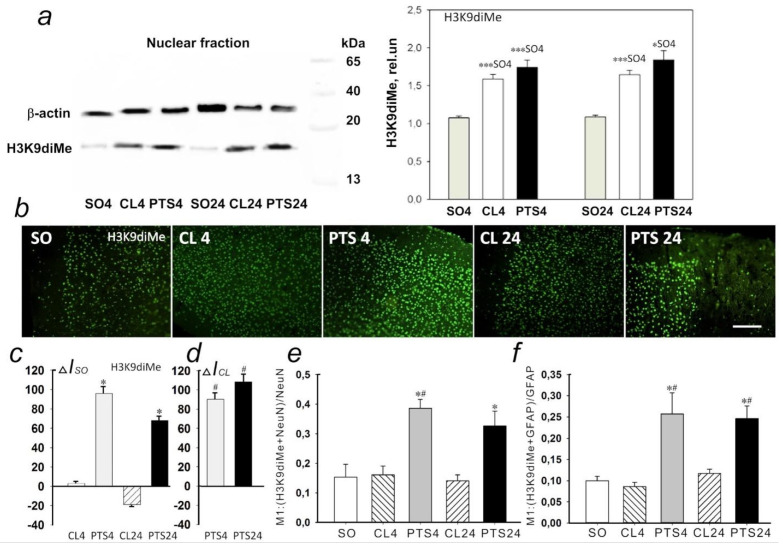
Expression of lysine 9 dimethylated histone H3 (H3K9diMe) in the penumbra and control samples of the cerebral cortex of sham-operated rats (SO) or the cerebral cortex in the contralateral hemisphere of the same animal (CL4 and CL24) at 4 and 24 h after photothrombotic stroke in the right hemisphere of the rat brain (PTS4 and PTS24). (**a**) Western blotting of H3K9diMe in the nuclear fraction of rat brain tissue. One Way ANOVA; M ± SEM, N = 8, * *p* < 0.05; *** *p* < 0.001 relative to sham-operated animals. (**b**) Immunofluorescence of H3K9diMe-positive cells in the cerebral cortex of sham-operated rats (SO), in the unirradiated contralateral cortex (CL4 and CL24) and in the penumbra at 4 and 24 h after photothrombotic stroke (PTS4 and PTS24). Scale section: 150 microns. (**c**) Percentage changes in the fluorescence intensity of H3K9diMe-positive cells in the penumbra and contralateral cortex at 4 or 24 h after PTS relative to that in sham-operated animals (ΔIso). (**d**) Percentage changes in the fluorescence intensity of H3K9diMe-positive cells in the penumbra at 4 or 24 h after PTS in the rat cerebral cortex relative to the contralateral cortex (ΔICL). ANOVA, *n* = 8, * *p* < 0.05 relative to sham-operated animals, # *p* < 0.05 relative to the contralateral hemisphere. (**e**,**f**) Average values of the M1 colocalization coefficient reflecting the proportion of pixels with a red signal (cellular marker, NeuN (**e**) or GFAP (**f**)) containing the green signal (H3K4me) in relation to the common signal from the red channel. M ± SEM, ANOVA, *n* = 8; * *p* < 0.05 relative to sham-operated animals; # *p* < 0.05, relative to the contralateral hemisphere. Rel.un—the ratio of the optical density of the strip of the protein studied to the optical density of the strip of the protein load marker (actin).

According to Western blotting data at both 4 h and 24 h after PTS the level of H3K9diMe in the penumbra significantly increased compared to the cortex of sham-operated animals by 62 (*p* < 0.001) and 69% (*p* < 0.05), respectively (Figure 10a). At the same time, in the control contralateral cortex of experimental rats, the level of H3K9diMe did not differ significantly from the penumbra, but significantly exceeded the level in the cortex of sham-operated animals.

These data are confirmed by the results of immunofluorescence microscopy (Figure 10b). Immunofluorescence of H3K9diMe-positive cells in the penumbra at PTS4 and PTS24 exceeded that in the cerebral cortex of SO rats by 90 and 60%, respectively (Figure 10c), and in the unirradiated contralateral cortex of the same animals (CL4 and CL24) approximately by 90 and 100%, respectively (Figure 10d). At the same time, the coefficient M1 of H3K9diMe colocalization with the neuron marker increased by a factor of 2.4 (Figure 10e) and by a factor of 2 with the astrocyte marker GFAP (Figure 10f). Thus, the level of H3K9diMe increased in the penumbra cells relative to the cerebral cortex of SO rats and the contralateral cortex of the same animals at PTS4 and PTS24. This increase occurred in both neurons and astrocytes.

**Figure 11:**
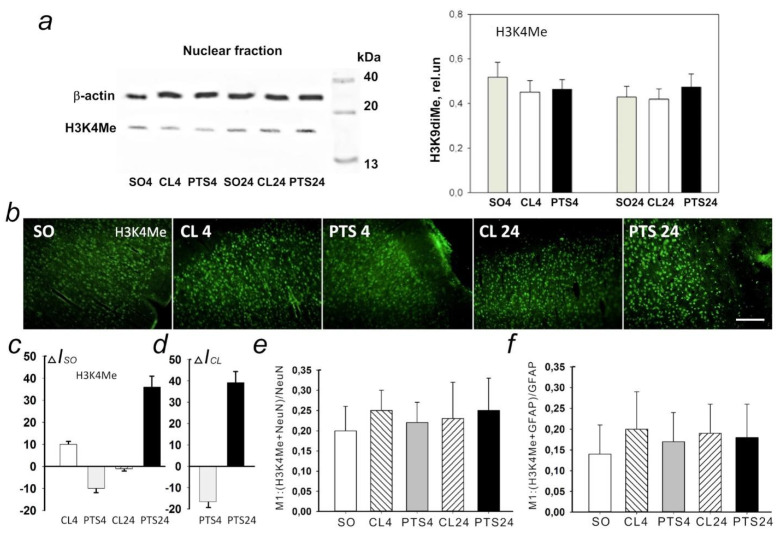
Expression of histone H3 monomethylated for lysine 4 (H3K4Me) in the penumbra and control samples of the cerebral cortex of sham-operated rats (SO) or cerebral cortex in the contralateral hemisphere of the same animal (CL4 and CL24) 4 and 24 h after photothrombotic stroke in the right hemisphere of the rat brain (PTS4 and PTS24). (**a**) Western blotting of H3K4Me in the nuclear fraction of rat brain tissue. One Way ANOVA; M ± SEM, *n* = 8. (**b**) Immunofluorescence of H3K4Me-positive cells in the cerebral cortex of SO rats, in the unirradiated contralateral cortex (CL4 and CL24) and in the penumbra at PTS4 and PTS24. Scale section: 150 microns. (**c**) Percentage changes in the fluorescence intensity of H3K4me-positive cells in the penumbra and contralateral cortex at PTS4 and PTS24 relative to that in SO animals (ΔIso). (**d**) Percentage changes in the fluorescence intensity of H3K4Me-positive cells in the penumbra at PTS4 and PTS24 in the rat cerebral cortex relative to the contralateral cortex (ΔICL). One Way ANOVA, *n* = 8. (**e**,**f**) Average values of the M1 colocalization coefficient reflecting the proportion of pixels with a red signal (cellular marker, NeuN (**e**) or GFAP (**f**)), containing a green signal (H3K4Me), in relation to the common signal from the red channel. M ± SEM, ANOVA, *n* = 8; Rel.un—the ratio of the optical density of the strip of the protein studied to the optical density of the strip of the protein load marker (actin).

Western blotting (Figure 11a) and immunofluorescence microscopy (Figure 11b) did not reveal significant changes in H3K4Me expression in the penumbra at 4PTS4 and PTS24 as compared with the contralateral cortex of the same rats and with the cerebral cortex of SO animals.

Expression of Н3К4Ме was observed in the nuclei of neurons and in a small number of astrocytes in the rat cerebral cortex. Colocalization of the protein with cellular markers in the penumbra did not change significantly compared to the control groups (Figure 11e,f).

### 2.5. DNMT1, SUV39H1, and G9a in Apoptotic Penumbra Cells

**Figure 12:**
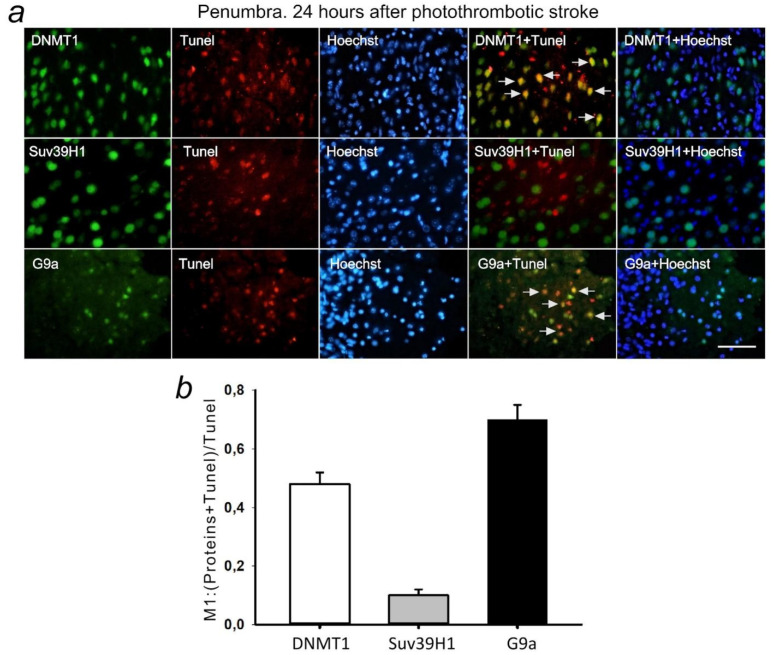
(**a**) Immunofluorescence of DNMT1, Suv39H1, G9a, and TUNEL-positive apoptotic cells, nuclear chromatin marker Hoechst 33342, and image overlay in the penumbra 24 h after photothrombotic stroke in the rat cerebral cortex. The scale bar is 100 μm. Cells containing DNMT1 or G9a co-localized with the TUNEL-positive apoptotic cells (shown by white arrows). (**b**) Coefficient M1 of DNMT1, Suv39H1, or G9a co-localization with TUNEL in penumbra at 24 h after photothrombotic stroke. M ± SEM; *n* = 7.

Double fluorescent staining of penumbra tissue sections with antibodies against the studied epigenetic proteins and the apoptosis marker TUNEL showed antibodies against DNMT1 and G9a, but not SUV39H1 colocalized with the nuclei of apoptotic cells at 24 h after PTS (Figure 12). This indicates the possible involvement of DNMT1 and G9a proteins in PTI-induced apoptosis.

### 2.6. Inhibitory Analysis

**Figure 13:**
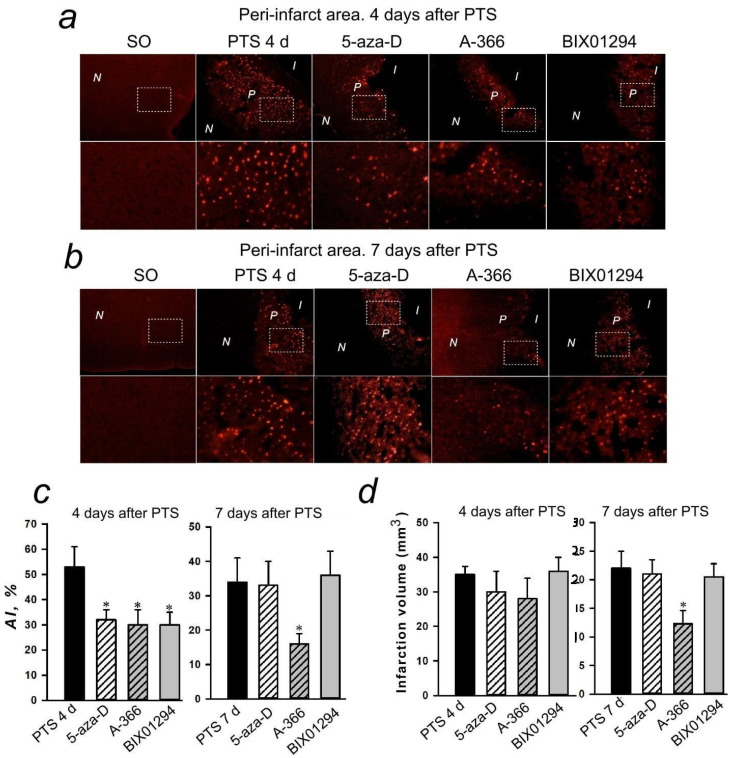
Apoptosis in the peri-infarct (P) area induced by photothrombotic stroke (PTS) in the mouse cerebral cortex. (**a**,**b**) Typical images of cortical regions stained with TUNEL (red fluorescence of apoptotic cells) at 4 days (**a**) and 7 days (**b**) after PTS. P-peri-infarct area, I-infarct area, N-normal tissue area. Control: sham-operated animals (SO). Experimental groups: The cerebral cortex of mice injected by various inhibitors. Inhibitors: 5-aza-2′-deoxycytidine (5-aza-D), a selective DNMT1 inhibitor; A-366 and BIX01294, a nonspecific inhibitor of Suv39H1 and G9a. Scale bar 200 μm, and the immunofluorescence of TUNEL in the peri-infarct (P) area at bigger magnification (Scale bar 50 μm) (**c**) Changes in the apoptotic index (AI, %) in the mice of experimental groups at 4 and 7 days after PTS introduction of various inhibitors. (**d**) The effects of inhibitors of DNMT1 and methyltransferases Suv39H1 and G9a on the infarction core volume in the mouse brain at 4 and 7 days after PTS. Mean values of the infarction core volume (mm^3^) in the control group (PTS without inhibitors) and in the experimental groups (administration of inhibitors). Scale bar 1 cm. One Way ANOVA; M ± SEM; *n* = 7–10. * *p* < 0.05 compared to the PTS in the absence of inhibitors.

The nuclei of apoptotic cells are localized in a band about 1–1.5 mm wide on sections of the cerebral cortex of mice subjected to PTS. The apoptosis was not observed from the left and right of this band where the infarction nucleus and normal tissue are located. The effect of inhibitors on the level of apoptosis in the penumbra was studied at 4 days after PTS (Figure 13c) where 5-aza-2′-deoxycytidine, A-366, or BIX01294 significantly reduced the percentage of apoptotic cells in the penumbra. This effect persisted only for A-366 at 7 days after PTS. (Figure 13d).

The administration of inhibitors of the studied epigenetic proteins to mice had no effect on the volume of infarction of the nervous tissue in the brain of mice at 4 days after PTS. However, in the group of mice injected with A-366 (but not other inhibitors) the infarction volume was reduced compared to the control group (PTS without inhibitor) at 7 days after PTS (Figure 13).

## 3. Discussion

DNA methyltransferase DNMT1 attaches methyl groups to one of the DNA strands where the complementary strand is methylated after cell repair or division. This study has shown that the level of DNMT1 in the nuclear and cytoplasmic fractions of the penumbra tissue did not change at 4 h after PTS in the rat cerebral cortex but increased at 24 h after PTS. Immunofluorescence microscopy confirmed an increase in the level of DNMT1 in the penumbra at 24 h after PTS. The colocalization of DNMT1 with markers of neurons (NeuN) and astrocytes (GFAP) was increased. As NeuN selectively labels the nuclei of neurons, and GFAP stains the bodies of astrocytes, including processes, these data explain the results of immunoblotting. It is likely that the increase in the level of DNMT1 in the nuclear fraction of the penumbra was due to increased expression in the nuclei of neurons, and an increase in the level of DNMT1 in the cytoplasmic fraction was due to the expression of this protein in the body and processes of astrocytes.

The investigated interval, 24 h, when DNMT1 is expressed, corresponds to the acute post-stroke period. According to data published previously [21,22] DNMT1 expression and subsequent global DNA methylation that suppresses protein biosynthesis increased at 16–24 h after cerebral ischemia and reperfusion. A number of authors suggest a relationship between DNMT1 activation, DNA methylation, and gene expression disorders with neuronal death [21,22,23,24,25]. It is possible ischemia and reperfusion, which cause oxidative damage to DNA, stimulate DNA repair in subsequent hours that necessitates the maintenance of DNA methylation and DNMT1 expression. In this case a correlation is realized between DNA damage in the ischemic brain, DNA methylation, and apoptosis. Our study showed colocalization of DNMT1 with apoptotic cell nuclei in PTS-induced penumbra. Inhibition of DNMT1 by 5-aza-2′-deoxycytidine protected cells of PTS-induced penumbra from apoptosis. The inhibition, but not complete knockout, of DNMT1 protected postmitotic neurons from ischemic damage [21,22].

In our previous study, we have shown that inhibition of DNA methylation by 5-azacytidine and 5-aza-2′-deoxycytidine (decitabine) reduced the level of PDT-induced necrosis of glial cells, but not neurons, by the factor of 1.3 and 2.0, respectively, and did not significantly influence apoptosis of glial cells [26].

This suggests the involvement of DNMT1 in apoptosis of penumbra cells after ischemic stroke. Therefore, the inhibition of DNA methylation may be a potential therapeutic strategy for the treatment of stroke.

Histone methyltransferases SUV39H1 and G9a are localized in cell nuclei where lysines are methylated in histones H3 and H4. It is known that methylation of lysines 9 and 27 in histone H3, as well as lysine 20 in histone H4, globally suppresses transcription while methylation of lysine 4 in histone H3 often correlates with transcriptional activity. In control samples, SUV39H1 and G9a were detected by immunoblotting in the nuclear fraction of the rat cerebral cortex, while they were practically absent in the cytoplasm of the cells. An increase in the level of SUV39H1 in the penumbra was found at 24 h after PTS and G9a was overexpressed at 4 and 24 h after PTS. In our experiments, photothrombotic stroke in the rat cerebral cortex caused an increase in the level of H3K9diMe, but not H3K4Me, in neurons and astrocytes of the penumbra 4 h after exposure, and it lasted for at least 24 h. G9a plays a major role in histone H3 lysine 9 methylation [27]. It is possible that G9a played a key role in the dimethylation of histone H3 (H3K9Me2). However, it should be noted that the main function of SUV39H1 is not di-, but trimethylation of H3K9. In previous experiments, the H3K9Me3 level was not estimated, which clearly must be done in the future to clarify the real role of SU39H1.

We observed colocalization of G9a with apoptotic cell nuclei in PTS-induced penumbra. G9a inhibitors A-366 and BIX01294 protected penumbra cells from apoptosis and reduced the volume of PTS-induced cerebral infarction. This suggests the involvement of G9a in apoptosis of penumbra cells after ischemic stroke.

## 4. Methods

### 4.1. Animals

The experiments were carried out on adult male rats weighing 200–250 g. Experiments with enzyme inhibitors were carried out on male outbred CD-1 mice at the age of 14–15 weeks weighing 20–25 g. The animals were kept under standard conditions with free access to water and food at 22–25 °C, 12-h light/dark schedule, and an air exchange rate of 18 shifts per hour. Body temperature was monitored with a rectal thermometer and maintained within 37 ± 0.5 °C using an electric mat. International, national, and institutional guidelines for the care and conduct of animal experiments were followed. The animal protocols were evaluated and approved by the Animal Care and Use Committee of the Southern Federal University.

### 4.2. Photothrombotic Stroke Model

Rats were anesthetized by intraperitoneal administration of telazol (50 mg/kg) and xylazine (10 mg/kg) [28]. For mice, 25 mg/kg telazol and 5 mg/kg xylazine were used for anesthesia [29].

The PTS procedure has been previously described [30]. Briefly, PTS in the rat cerebral cortex was induced by laser irradiation (532 nm, 60 mW/cm2, Ø 3 mm, 30 min) of a part of the sensorimotor cortex of the rat brain after intravenous injection of the Rose Bengal photosensitizer (R4507, Sigma, Rehovot, Israel; 20 mg/kg). For PTS in the cerebral cortex of mice, Rose Bengal (15 mg/mL) was injected intraperitoneally (10 μL/g). A section of the mouse skull was freed from the periosteum in the area of the sensorimotor cortex (2 mm lateral to the bregma) and this part of brain was irradiated with a laser (532 nm, 200 mW/cm2, Ø 1 mm, 15 min) 5 min after photosensitizer was applied. The sham-operated animals that underwent the same operations, but without the introduction of a photosensitizer, were used as controls.

### 4.3. Immunofluorescence Microscopy Analysis

For immunofluorescence microscopy, the rats were anesthetized and transcardially perfused with 10% formalin 4 or 24 h after PTS, as described previously [30]. Briefly, control samples were from the contralateral cortex of the same animals (CL4 and CL24, respectively) or the cerebral cortex of sham-operated rats (SO4 and SO24). The brain was fixed in formalin overnight and incubated for 48 h in 20% sucrose in phosphate buffer (PBS) at 4 °C. Frontal sections of the brain with a thickness of 20 μm were prepared using a Leica VT 1000 S vibratome (Freiburg, Germany). They were frozen in 2-methylbutane and stored at −80 °C. After thawing, the sections were washed with PBS. The sections were then incubated overnight at 4 °C in the same solution with primary rabbit antibodies (SigmaAldrich, Rehovot, Israel): anti-DNMT1 (D4567, 1:500); anti-G9a (09–071, 1:100), anti-SUV39H1 (AV32470, 1:500) and anti-NeuN mouse antibody (MAB377; 1:1000) or anti-GFAP (SAB5201104; 1:1000), anti-histone H3, dimethylated at lysine 9 (anti-H3K9diMe; D5567, 1:500), and anti-histone H3, methylated at lysine 4 (anti-H3K4Me; M4819, 1:500). After washing in PBS, the sections were incubated for 1 h with fluorescently labeled secondary anti-rabbit CF488A (SAB4600045, 1:1000) or anti-mouse CF555 (SAB4600302, 1:1000) antibodies. Non-specific antibody binding was blocked by 5% BSA with 0.3% Triton X-100 (1 h, 20–25 °C). Sections were mounted on glass slides in 60% glycerol/PBS. Negative control: no primary antibodies. Sections were analyzed using an Eclipse FN1 microscope (Nikon, Tokyo, Japan).

In most of the experiments, fluorescent images were studied in the central region of the penumbra, at a distance of 0.3–0.7 mm from the border of the infarction nucleus. The quantitative assessment of fluorescence was carried out on 10–15 images of experimental and control preparations obtained with the same settings of a digital camera. The average fluorescence intensity in the area occupied by cells was determined in each image using the ImageJ software (http://rsb.info.nih.gov/ij/, accessed on 20 October 2021). The corrected fluorescence intensity of cells I (CTCF), proportional to the level of protein expression, was calculated as: I = Ii − Ac * Ib; where Ii is the integral fluorescence intensity, Ac is the cell area, and Ib is the average background fluorescence [30]. Threshold values remained constant for all images. The relative changes in the fluorescence of cells in the penumbra compared to the control cortex, ΔI, were calculated as: ΔI = (Ipen − Ic)/Ic, where Ipen is the average fluorescence intensity in the penumbra, and Ic is the average fluorescence intensity in the control samples.

Additionally, using ROI Manager tools in ImageJ, the immunofluorescence of DNMT1, Suv39H1, and G9a proteins in neuronal nuclei was assessed. Immunofluorescence data of proteins in cell nuclei were normalized to Hoechst 33342 fluorescence. In addition, the number of cells expressing the protein under study was calculated per 100 cells.

Protein colocalization with the neuronal marker NeuN or the astrocyte marker GFAP was assessed using Image J (http://rsb.info.nih.gov/ij/, accessed on 20 October 2021) with the JACoP plugin. On RGB images (1280 × 960), the Manders coefficient M1 reflects the proportion of pixels containing red (cell markers or TUNEL staining) and green (proteins) signals in the total signal recorded in the red channel. In each area of the brain, three fields of vision were analyzed in 7–10 rats. Statistical processing was performed according to OneWay ANOVA. Results are presented as M ± SEM.

### 4.4. Apoptosis Analysis

Apoptotic cells were visualized by the TUNEL method (TdT-mediated dUTP-X nick end labeling) using the In Situ Cell Death Detection Kit, TMR red (# 12156792910, Roche, Mannheim, Germany) (red fluorescence) as described previously [30]. Briefly, sections were incubated at 37 °C with a primary antibody against the protein under study (green fluorescence), washed, treated with reagents from this kit, and incubated for 1 h with a secondary antibody Anti-Rabbit CF488A (SAB4600045, 1:1000) (green fluorescence) and with the Hoechst cell nucleus marker 33342 (10 μg/mL, blue fluorescence). The apoptotic coefficient (AI) was calculated as a percentage as:

AI = (number of TUNEL-positive cells/total number of cells stained with Hoechst 33342)

The analysis was performed on 3 images for each of 7–9 animals in the group.

### 4.5. Immunoblotting

The immunoblotting procedure has been previously described [30]. Briefly, the rats were anesthetized and decapitated 4 or 24 h after PTS. The brain was removed and a section of the cortex corresponding to the infarction nucleus was removed on ice with a cylindrical knife Ø3 mm; with another knife Ø7 mm, a 2-mm ring was cut around the irradiation zone, approximately corresponding to the penumbra tissue (experimental sample). A similar control sample was excised in the unirradiated contralateral hemisphere or in the cerebral cortex of sham-operated rats. Pieces of tissue were homogenized on ice, quickly frozen in liquid nitrogen, and then stored at −80 °C. After thawing and centrifugation using the CelLytic NuCLEAR Extraction Kit (SigmaAldrich), the cytoplasmic and nuclear fractions were isolated from the homogenates. The total supernatant was used as the cytoplasmic fraction, in which the nuclear protein histone H3 was practically not detected. Primary rabbit antibodies (all SigmaAldrich) were used in the experiments: anti-DNMT1 (D4567, 1:500), anti-G9a (09-071, 1:500), anti-SUV39H1 (AV32470, 1:500), and mouse anti-β-actin antibody (A5441, 1:5000). Secondary antibodies (all SigmaAldrich): anti-Rabbit IgG-Peroxidase (A6154, 1:1000) and anti-Mouse IgG-Peroxidase (A4416, 1:1000).

### 4.6. Inhibitors

The study investigated the putative neuroprotective effect of the following inhibitors of epigenetic proteins were used: DNMT1 (5-azacytidine or decitabine (0.2 mg/kg, 1 per day, 7 days) [31,32,33], histone methyltransferases SUV39H1 and G9a (BIX01294 (0.5 mg/kg, once a day, 7 days) [34], and A-366 (2 mg/kg, once a day, 7 days) [35]. They were dissolved in dimethyl sulfoxide (DMSO) and then diluted in sterile saline. The final concentration of DMSO was 5%. All inhibitors were administered 1 h after PTS. Mice were decapitated 4 and 7 days after PTS and 1 h after the last injection of drugs to study the level of apoptosis in the peri-infarction area and the volume of infarction.

### 4.7. Determination of the Volume of Infarction

To determine the volume of infarction at different times after PTS, brain slices of mice were stained with 2,3,5-triphenyltetrazolium chloride (TTX; T8877, Sigma). For this, the mice were anesthetized and decapitated, the brains were quickly removed and placed in a pre-chilled adult mouse brain matrix (J&K Seiko Electronic Co., Ltd., DongGuan City, China). The matrix with brain tissue was transferred to a freezer (−80°C) for 3–5 min and sliced 2 mm thick. These sections were stained with 1% TTX for 30 min in the dark at 37 °C. Using the ImageJ image analysis software (http://rsb.info.nih.gov/ij/, accessed on 20 October 2021), the areas of infarction zones in each section were measured, summed up, and multiplied by the section thickness (2 mm).

## 5. Conclusions

The data obtained show that DNA methyltransferase DNMT1 and histone methyltransferase G9a can be potential protein targets in ischemic penumbra cells, and their inhibitors, such as 5-aza-2′-deoxycytidine, A-366, and BIX01294, are potential neuroprotective agents capable of protecting penumbra cells from postischemic damage to the cerebral cortex. The protective effect of these substances should be studied in more detail in the future. Compound A-366 had the most persistent protective effect on the mouse cerebral cortex after photothrombotic stroke. Therefore, it can be recommended as a promising anti-ischemic neuroprotector.

## Data Availability

Data is available on demand to corresponding author Demyanenko S. V.
